# Multidimensional self-rating biological rhythm disorder and its association with depression and anxiety symptoms among adolescents aged 11–23 years: a school-based cross-sectional study from China

**DOI:** 10.1186/s12888-022-04354-8

**Published:** 2022-11-14

**Authors:** Xin Zeng, Yiyang Chen, Qian Zhang, Yexin Jin, Yalin Song, Kunyu Xue, Hao Lou, Ran Li, Xiaomin Lou, Xian Wang

**Affiliations:** 1 College of Public Health, Zhengzhou University, No.100 Science Avenue, Zhengzhou, 450001 Henan P.R. China; 2 Zhongmu County Center for Disease Control and Prevention, No. 1106, West Qingnian Road, Zhengzhou, 451450 Henan P.R. China; 3 Department of Nosocomial Infection Management, the First Affiliated Hospital of Zhengzhou University, Zhengzhou, 450052 Henan P.R. China; 4 Zhengzhou Station for Students’ Health, Zhengzhou, 450007 Henan P.R. China

**Keywords:** Biological rhythm disorder, Depression, Anxiety, Adolescents, China

## Abstract

**Background:**

Depression and anxiety are topical concerns worldwide, especially among adolescents. Besides, biological rhythm disorder as a candidate mechanism for mood disorders is highly prevalent, but relevant research among adolescents in China is presently limited. We conducted the present study to investigate the distribution of multi-dimensional self-rating biological rhythm disorder and the association of self-rating biological rhythm disorders with depression and anxiety symptoms among Chinese adolescents in different academic stages.

**Methods:**

In the cross-sectional study, 3693 students aged 11–23 from Zhengzhou City, Henan Province, China were included. The Patient Health Questionnaire (PHQ-9) and General Anxiety Disorder (GAD-7) were used to evaluate symptoms of depression and anxiety, respectively. Additionally, the Self-Rating of Biological Rhythm Disorder for Adolescents (SBRDA) was used to assess status of biological rhythm disorders. Multivariate logistic regression was developed to explore factors potentially associated with symptoms of depression and anxiety stratified by academic stages.

**Results:**

Among all participants, 44.14 and 36.15% suffered from depression and anxiety symptoms, respectively. On average, participants scored 74.66 ± 19.37 on the measure of total biological rhythm disorder. Adjusted for demographic confounding factors, the logistic regression analysis showed higher scores of total biological rhythm disorder were associated with more severe depression (OR = 14.38, 95%CI: 11.38–18.16) and anxiety symptoms (OR = 11.63, 95%CI: 9.14–14.81). The similar results were also found in the stratified analysis by academic stages.

**Conclusions:**

Self-rating biological rhythm disorders are significantly associated with depression and anxiety symptoms among adolescents. Discrepancy across academic stages should also be taken into account in establishing public health strategies.

**Supplementary Information:**

The online version contains supplementary material available at 10.1186/s12888-022-04354-8.

## Introduction

Depression and anxiety are major public health problems of widespread global concern and are among the top causes of disease and disability among adolescents [1]. Seriously, the novel coronavirus disease 2019 (COVID-19), which has swept large parts of the globe, has aggravated the prevalence of depression and anxiety in all populations [2]. According to recent WHO data, the global detection rates of anxiety and depression soared by a huge 25% in the first year of the COVID-19 pandemic [3]. Recent studies have shown that COVID-19 exerts detrimental effects on adolescents particularly [4, 5]. Therefore, confronting the COVID-19 pandemic, it is necessary to pay attention to the mental disorders of adolescents such as depression and anxiety. However, the underlying pathophysiological mechanisms of mood syndromes lacks clear delineation, which is a major barrier to prevention and optimized treatments. Biological rhythm disorder is a candidate mechanism for mood disorders with genetic, behavioral, and neurobiological links [6].

Biological rhythm refers to cyclical variations in physiological and behavioral functions, comprising the sleep-wake cycle, social activity, eating pattern, and other important bodily functions, modulated by a circadian clock. Almost all organisms have an endogenous rhythm pattern that circulates daily (approximately 24 hours) to accommodate the 24-hour light-dark cycle [7]. Sleep is largely regulated by the light-dark cycle and relevant circadian rhythms. Previous researches have revealed that sleep rhythm disorder is associated with worse clinical outcomes [8, 9]. For instance, a recent study reported that sleep rhythm disorder could affect the symptom manifestation and pathogenesis of digestive disorders [10]. Studies regarding biorhythm disorders have mainly focused on the sleep-wake cycle. However, in a broader sense, the role of multidimensional biological rhythm, including eating habits rhythm, activity rhythm, and so on, is less well understood. Timed meals play a significant role in synchronizing the peripheral circadian rhythms in humans [11]. Original studies and a related review have suggested that irregular eating patterns (such as eating jetlag between weekdays and weekends, mistimed eating, etc.) have deleterious consequences on metabolic health [12–15]. In terms of the rhythm of social behavior in daily life, namely activity rhythm, a related therapy called social rhythm therapy (SRT) was researched to promote the stabilization of daily behavior and thus minimize the effects of circadian rhythm disorder [16]. In addition, the use of the digital media in contemporary society has also profoundly affected biological rhythms. There is evidence that excessive screen time during digital media use can affect sleep-related factors, thereby leading to relevant rhythm disorders [17, 18]. Of note, the circadian system of adolescents will continue to change until adulthood [19] and is easily influenced by external environmental factors, so more attention should be paid to the population.

Previous studies have confirmed the association between biological rhythm disorders and mood disorders. Nevertheless, a majority of researches on biological rhythms have solely focused on sleep. Individuals with an evening chronotype and high social jetlag are at increased risk of experiencing emotional problems such as depression and so on [20–22]. Researchers have assessed biological rhythm disorders from a multidimensional perspective using the Biological Rhythms Interview of Assessment in Neuropsychiatry (BRIAN), a validated instrument that evaluates the four main areas related to biological rhythm disorders, namely sleep, social rhythms, activity, and eating pattern [23]. For example, a large multi-center research conducted in Spain, Brazil and Canada showed that biological rhythm disorder may act as an independent predictor of poor psychosocial functioning in bipolar disorder [9]. Some studies among individuals aged 18–24 demonstrated higher levels of biological rhythm disorders in individuals with depression [24] or bipolar disorder [25]. However, there is a gap in the literature about biological rhythm assessing from multiple dimensions among adolescents (particularly in 11–17 years), especially in China. Notably, although BRIAN developed in 2009 had the advantage of multi-dimensional assessment, it failed to consider the emerging risk factors related to biorhythm disorders such as digital media use. One survey found that, during the COVID-19 pandemic, adolescents were more likely to spend much time using digital media to study online, play games, and chat with friends [26]. Thus, the overexposure to the digital media should also be considered in assessing biorhythm disorders.

Since adolescence is a rapidly changing phase [27], the effects of biorhythm disorders on emotion and behavior may be quite different across academic stages. Therefore, we conducted this study with the latest questionnaire scale among Chinese adolescents stratified by academic stages, aiming to investigate the distribution of multi-dimensional self-rating biological rhythm disorder (sleep rhythm, activity rhythm, eating habits rhythm, and digital media use) in Chinese adolescents, as well as the association of self-rating biological rhythm disorders with depression and anxiety symptoms across academic stages. To be noted, the definition of adolescence was prolonged in our study.

## Method

### Sampling and procedure

A cross-sectional survey was conducted among adolescents ranging from junior high school to university in Zhengzhou City, Henan Province, China, from September 2021 to December 2021. We selected one urban junior high school, one urban senior high school, one urban vocational high school, one rural junior high school, one rural senior high school, one rural vocational high school, and one provincial university. Using a cluster sampling method and by considering one class as a unit, we randomly selected classes from three grades (four grades for university) such that the number of students in each grade was approximately 200. All students in the selected classes were eligible to participate in the survey except for those with serious organic diseases or serious psychological disorders. For some reasons, the sampling of the third grade of the urban vocational high school was not completed, so a total of 4351 students were surveyed. The sampling process can be seen in Fig. S1. After excluding 658 students who submitted incomplete questionnaires with missing important data, the remaining 3693 participants were ultimately included in the current analysis. There was no difference in the basic characteristics between the included population and the initial population (Table S1).

Informed consent was obtained prior to participation for all participants over 16 years of age and for legal guardians of participants under 16 years of age. After describing the purpose and procedures of the study, trained investigators handed out the questionnaires to the participants and instructed them to fill out the questionnaires independently. Investigators were on hand at each site to help participants with any confusion or questions they had regarding the structured questionnaire. The study was approved by the Zhengzhou University Ethics Committee (ZZUIRB 2021–94).

### Measuring sociodemographic data

Sociodemographic data for participants were collected including age, gender (male or female), grade (junior high school, vocational high school, senior high school or university), residential areas (rural or urban), the only child status (yes or no), parents’ education levels (below the elementary school, elementary school, junior high school, senior high school or technical school, or junior college or above), self-perceived family economy (under moderate, moderate or over moderate), self-perceived study burden (low, medium, high) and the number of close friends (0, 1–2, 3–5 or > 5).

### Self-rating biological rhythm disorders assessment

The Self-Rating of Biological Rhythm Disorder for Adolescents (SBRDA) [28] was used to assess the current status of biological rhythm disorders in adolescents. A total of 29 items were assessed, with four dimensions considered: sleep rhythm, activity rhythm, eating habits rhythm and digital media use. The adolescents’ biological rhythm disorders were assessed using a five-point Likert scale, with scores ranging from one to five, corresponding to “completely inconsistent”, “basically inconsistent”, “somewhat consistent”, “basically consistent” and “completely consistent” respectively. Sleep rhythm scores range from 6 to 30, activity rhythm scores range from 7 to 35, eating habits rhythm scores range from 8 to 40, digital media use scores range from 8 to 40 and total rhythm disorder rhythm scores range from 29 to 145. The higher the questionnaire score, the more severe the self-rating biological rhythm disorders. According to Kelly’s recommendation [29], those who scored lower than 27% were classified in the low score group, while those who scored higher than 73% were classified in the high score group and the rest were classified in the middle score group.

### Depression and anxiety symptoms assessment

Depression level was assessed by the Patient Health Questionnaire-9 (PHQ-9) [30], a measure that assesses the severity of depression. The PHQ-9 asks participants how frequently they have been bothered by depression symptoms in the past 2 weeks. The nine-items are rated on a four-point Likert scale from zero to three (0 = not at all, 1 = several days, 2 = more than half of the days, 3 = nearly every day). The total score was calculated by adding the scores for each question, which ranged from 0 to 27, with higher scores indicating more severe depression symptoms. Anxiety level was assessed by the 7-item Generalized Anxiety Disorder (GAD-7) [31], a measure that assesses the severity of anxiety disorders within the past 2 weeks. Similar to PHQ-9, each item was scored from 0 (not at all) to 3 (nearly every day). In the current study, samples whose total scores > 4 in PHQ-9/GAD-7 were defined as suffering from depression/anxiety symptoms respectively.

### Statistical analysis

Statistical analyses were performed with SPSS 21.0. Descriptive analyses were used to show the demographic information of the sample. In sensitivity analysis, chi-square tests were used to compare differences in basic demographic characteristics between the included samples and the initial samples. Differences in demographics, total and each dimension of self-rating biological rhythm disorder scores, depression and anxiety symptoms among groups were tested using chi-squared test or Kruskal-Wallis H test. Additionally, multivariable logistic regression was used to explore the associations between self-rating biological rhythm disorders and depression and anxiety symptoms. Partial correlation analyses were used to explore bivariate relationships, adjusting for confounding factors. The logistic regression models in this study were conducted in total and stratified population of different academic stages. Models were controlled for age, gender, residential areas, parents’ educational levels, the only child status, self-perceived family economy, self-perceived study burden, and the number of close friends. Odds ratios (ORs) and their 95% confidence intervals (CIs) were calculated. The significance level was set at 0.05.

## Results

### Demographics of participants and the distribution of self-rating biological rhythm disorders

As displayed in Tables 1, 3693 students aged 11–23 years old (mean ± SD:16.30 ± 2.33) were included in our study. On average, the participants scored 74.66 ± 19.37(mean ± SD) on the measure of total biological rhythm disorder, and scored 18.37 ± 4.69 on the measure of sleep rhythm, 15.88 ± 4.89 on the measure of activity rhythm, 20.91 ± 7.00 on the measure of eating habits rhythm and 19.50 ± 7.42 on the measure of digital media use (Table S2). Students with high study burden, low self-perceived family economic status or a low number of close friends showed a higher proportion of high score group (*p* < 0.01). Moreover, compared to male students, female students showed a higher proportion of high score group (*p* < 0.01) (Table 1). In addition, distributions of four dimensions of self-rating biological rhythm disorder among different demographic variables were shown in Table S3-S6.

**Table 1:** Sample characteristics stratified by self-rating biological rhythm disorder (total) (*N* = 3693)^a^

Variables	N (%)	Biological rhythm disorder (Total)			
		Low (%)	Middle (%)	High (%)	***χ***^***2***^ ***/ K-W***
**Gender**					
Male	1782 (48.25)	555 (31.14)	797 (44.73)	430 (24.13)	37.96**
Female	1911 (51.75)	431 (22.55)	912 (47.72)	568 (29.72)	
**Academic stage**					
Junior high school	1020 (27.62)	426 (41.76)	396 (38.82)	198 (19.41)	182.96**
Vocational high school	849 (22.99)	212 (24.97)	386 (45.47)	251 (29.56)	
Senior high school	1067 (28.89)	222 (20.81)	539 (50.52)	306 (28.68)	
University	757 (20.50)	16 (16.64)	388 (51.25)	243 (32.1)	
**Residential areas**					
Rural	1890 (51.18)	450 (23.81)	911 (48.2)	529 (27.99)	16.54**
Urban	1803 (48.82)	536 (29.73)	798 (44.26)	469 (26.01)	
**Only child status**					
No	3240 (87.73)	858 (26.48)	1509 (46.57)	873 (26.94)	1.04
Yes	453 (12.27)	128 (28.26)	200 (44.15)	125 (27.59)	
**Father’s education**					
Below elementary school	133 (3.60)	30 (22.56)	58 (43.61)	45 (33.83)	6.87
Elementary school	509 (13.78)	125 (24.56)	238 (46.76)	146 (28.68)	
Junior high school	1678 (45.44)	435 (25.92)	795 (47.38)	448 (26.7)	
Senior high school or technical school	855 (23.15)	241 (28.19)	391 (45.73)	223 (26.08)	
Junior college or above	518 (14.03)	155 (29.92)	227 (43.82)	136 (26.25)	
**Mother’s education**					
Below elementary school	300 (8.12)	61 (20.33)	154 (51.33)	85 (28.33)	14.36**
Elementary school	658 (17.82)	155 (23.56)	306 (46.5)	197 (29.94)	
Junior high school	1495 (40.48)	394 (26.35)	699 (46.76)	402 (26.89)	
Senior high school or technical school	793 (21.47)	238 (30.01)	354 (44.64)	201 (25.35)	
Junior college or above	447 (12.10)	138 (30.87)	196 (43.85)	113 (25.28)	
**Self-perceived family economy**					
Under moderate	649 (17.57)	115 (17.72)	310 (47.77)	224 (34.51)	59.92**
Moderate	2656 (71.92)	722 (27.18)	1236 (46.54)	698 (26.28)	
Over moderate	388 (10.51)	149 (38.4)	163 (42.01)	76 (19.59)	
**Self-perceived study burden**					
Low	183 (4.96)	70 (38.25)	65 (35.52)	48 (26.23)	70.59**
Medium	2205 (59.71)	649 (29.43)	1055 (47.85)	501 (22.72)	
High	1305 (35.34)	267 (20.46)	589 (45.13)	449 (34.41)	
**The number of close friends**					
0	73 (1.98)	19 (26.03)	27 (36.99)	27 (36.99)	25.68**
1–2	1000 (27.08)	239 (23.90)	460 (46.00)	301 (30.10)	
3–5	1768 (47.87)	448 (25.34)	844 (47.74)	476 (26.92)	
> 5	852 (23.07)	280 (32.86)	378 (44.37)	194 (22.77)	

*p < 0.05; **p < 0.01

^a^ 295 samples were missing information on SBRDA, 105 samples were missing information on PHQ-9 and GAD-7, 79 samples were missing information on age, 19 samples were missing information on gender, 9 samples were missing information on residential areas, 4 samples were missing information on self-perceived family economy, 26 samples were missing information on parents’ education, 78 samples were missing information on only child status, 26 were missing information on the number of close friends, 17 were missing information on self-perceived study burden

**Fig. 1:**
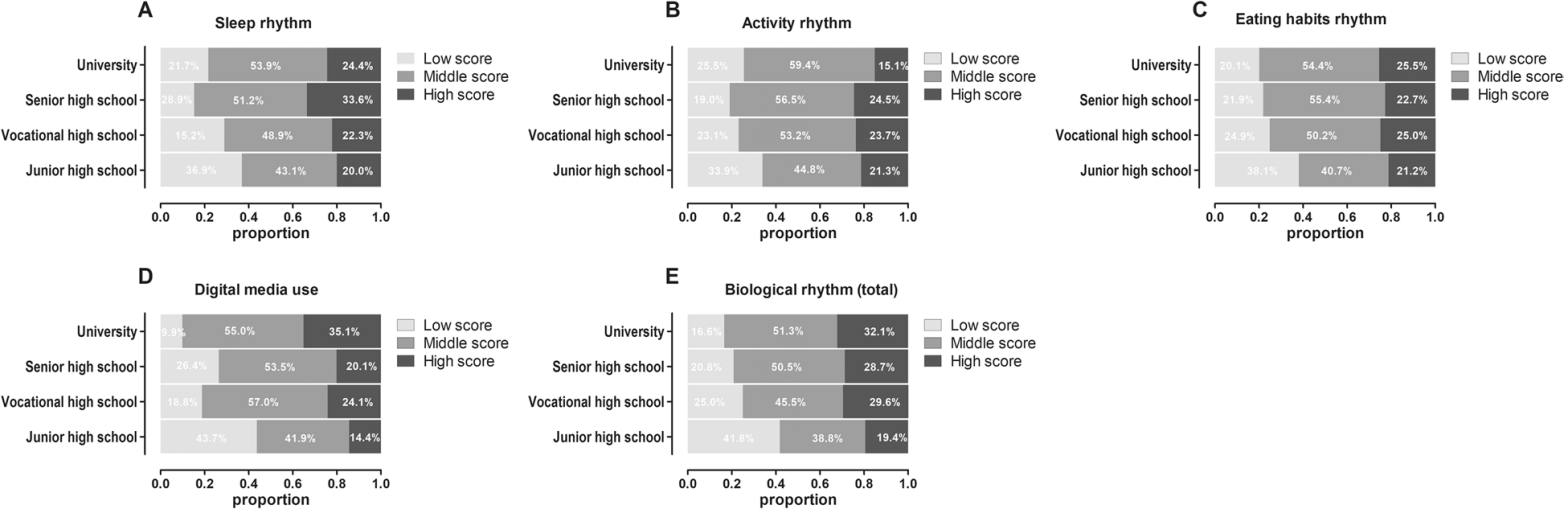
The proportion of the score group of total and each dimension of self-rating biological rhythm disorder in different academic stages

The proportions of score group of each dimension of self-rating biological rhythm disorders among different academic stages were shown in Fig. 1. Junior high school students overall showed lower proportion of middle and high score group of biological rhythm disorder in all dimensions than students in any other academic stage, meaning that junior high school students had the mildest disorders (Fig. 1A-E). Senior high school students had the most severe disorders in sleep rhythms and activity rhythms (Fig. 1A, B). Of note, vocational and senior high school students belong to the same academic phase, but their biological rhythm disorders were distributed differently. Both vocational high school and university students had more severe disorders in the digital media use dimension (Fig. 1D). In the total biological rhythm aspect, disorders were becoming more severe as the academic stage progressed. In the high school phase, vocational high school students preseted more severe biological rhythm disorders than senior high school students (Fig. 1E).

### Depression and anxiety symptoms with self-rating biological rhythm disorders

As shown in Table 2, depression symptoms were found in 44.14% (1630) of all the 3963 students; for males in 40.97% (730/1782), and females in 47.10% (900/1911). Likewise, anxiety symptoms were found in 36.15% (1335) of the total students, for males in 33.56% (598/1782), and females in 38.57% (737/1911). Overall, the positive rates of depression and anxiety symptoms were significantly higher with higher score group of total biological rhythm disorders (depression: 15.82, 43.83, 72.65%, respectively; anxiety: 12.58, 34.87, 61.62%, respectively). In particular, in activity rhythm aspect, the detection rates for depression and anxiety were both highest in the high score group among four dimensions (74.78% in depression, 63.43% in anxiety).

**Table 2:** Depression and anxiety symptoms with self-rating biological rhythm disorders

Variables		Depression symptoms			Anxiety symptoms		
		Without	With	***χ***^***2***^	Without	With	***χ***^***2***^
**N (%)**		2063 (55.86)	1630 (44.14)		2358 (63.85)	1335 (36.15)	
**Sleep rhythm**	**Low (%)**	717 (75.71)	230 (24.29)	276.37**	743 (78.46)	204 (21.54)	196.72**
	**Middle (%)**	992 (54.84)	817 (45.16)		1170 (64.68)	639 (35.32)	
	**High (%)**	354 (37.78)	583 (62.22)		445 (47.49)	492 (52.51)	
**Activity rhythm**	**Low (%)**	782 (83.37)	156 (16.63)	590.28**	812 (86.57)	126 (13.43)	465.43**
	**Middle (%)**	1081 (55.10)	881 (44.90)		1256 (64.02)	706 (35.98)	
	**High (%)**	200 (25.22)	593 (74.78)		290 (36.57)	503 (63.43)	
**Eating habits rhythm**	**Low (%)**	794 (80.53)	192 (19.47)	464.35**	828 (83.98)	158 (16.02)	384.16**
	**Middle (%)**	1003 (54.39)	841 (45.61)		1184 (64.21)	660 (35.79)	
	**High (%)**	266 (30.82)	597 (69.17)		346 (40.10)	517 (59.91)	
**Digital media use**	**Low (%)**	740 (76.84)	223 (23.16)	333.88**	781 (81.10)	182 (18.90)	257.93**
	**Middle (%)**	1040 (54.79)	858 (45.21)		1206 (64.55)	692 (35.45)	
	**High (%)**	283 (34.01)	549 (71.39)		371 (44.59)	461 (55.41)	
**Biological rhythm (Total)**	**Low (%)**	830 (84.18)	156 (15.82)	649.65**	862 (87.42)	124 (12.58)	519.17**
	**Middle (%)**	960 (56.17)	749 (43.83)		1113 (65.13)	596 (34.87)	
	**High (%)**	273 (27.35)	725 (72.65)		383 (38.38)	615 (61.62)	

*p < 0.05; **p < 0.01

### Adjusted associations of self-rating biological rhythm disorders with depression and anxiety symptoms

In the partial correlation analysis, a significant correlation was founded between the SBRDA score and depression and anxiety scale score after adjusting for confounders (*p* < 0.01) (Table S7).

**Fig. 2:**
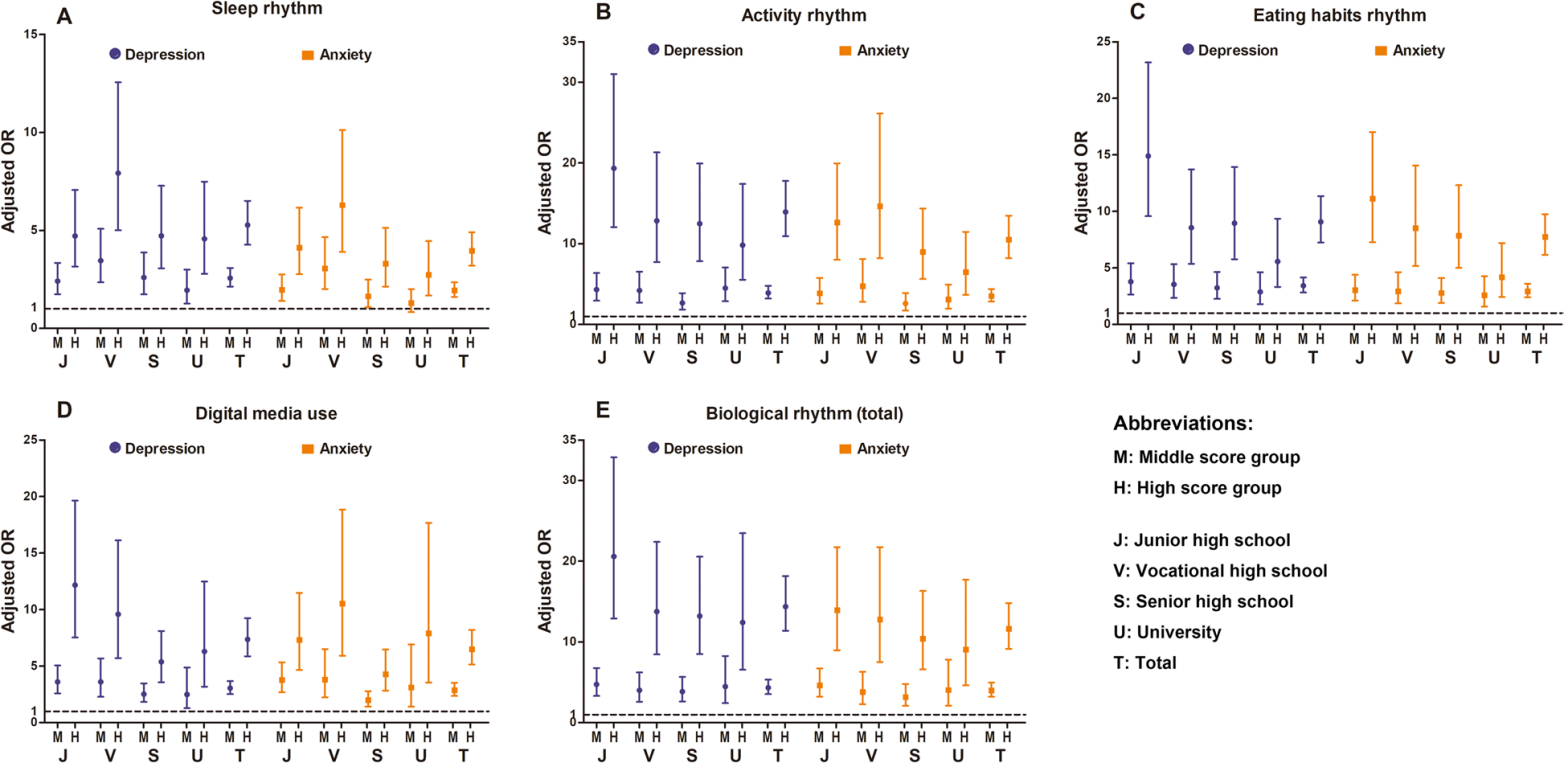
Multivariable-adjusted OR and 95% CI for depression and anxiety symptoms across different academic stages according to score group of total and each dimension of self-rating biological rhythm disorders (low score group as reference). Vertical lines represent the 95% CIs. Models were adjusted for age, gender, residential areas, parents’ educational levels, the only child status, self-perceived family economy, self-perceived study burden, and the number of close friends. Abbreviations: CI, confidence interval; OR, odd ratio; M, middle score group; H, high score group; J, junior high school; V, vocational high school; S, senior high school; U, university; T, total

As shown in Fig. 2 and Table S8-S9, among the total population, the regression results of total and each dimension of self-rating biological rhythm with anxiety and depression were statistically significant (*p* < 0.01). In the total biological rhythm level, compared to the low score group (reference group), the aOR of middle score group was 4.33 (3.53–5.32) for depression symptoms and 3.99 (3.19–4.98) for anxiety symptoms; the aOR of high score group was 14.38 (11.38–18.16) for depression symptoms and 11.63 (9.14–14.81) for anxiety symptoms. In the activity rhythm dimension, middle and high score were associated with greater risk of depression symptoms compared with low score, with aORs of 3.90 (95% CI 3.19–4.77), 13.93 (95% CI 10.92–17.77), respectively. Similar results were found for the risk of anxiety symptoms, with aORs of 3.53 (95% CI 2.84–4.38), 10.52 (95% CI 8.22–13.46), respectively. Similarly, the risk associated with other dimensions of self-rating biological rhythm and depression and anxiety symptoms was higher among students with middle and high score than those with low score.

Generally, in the stratified analysis of each academic stage, the association of each dimension of self-rating biological rhythm disorders with depression and anxiety symptoms was stronger among students with high score than those with middle or low score. In the total biological rhythm disorders aspect, junior high school students with high score were more likely to develop depression and anxiety symptoms than students with high score among other academic stages.

## Discussion

In this cross-sectional study among Chinese adolescents, a significant association was observed between total and four dimensions of self-rating biological rhythm disorders and depression as well as anxiety symptoms. These findings regarding depression symptoms were consistent with several previous studies. For example, a Brazilian study of 18–24 years old participants using the BRIAN scale found that adolescents with depression had higher BRIAN scores [32]. An Australian study using Hamilton Depression Rating Scale and Young Mania Rating Scale to assess depression symptoms also suggested that delayed and disturbed circadian rhythms may be associated with worse mental health [33]. Notably, limited studies explored association between self-rating biological rhythm disorders and anxiety symptoms, so we considered it as a complement in the present study. Furthermore, of four dimensions, activity rhythm disorder was found to be most associated with depression and anxiety symptoms, while sleep rhythm disorder was found to be least. The result was similar with a cross-sectional study investigating the relationship between subjective changes in biological rhythms and worsening of depression symptoms in 83 women from pregnancy to the postpartum period. The researchers found that subjective disruptions in social, eating, and activity rhythms, as evaluated by the BRIAN scale, were more strongly associated with depression symptoms than sleep rhythm [34]. Disruptions in other rhythms such as activity, eating habits rhythm may have occluded the impact of sleep disorder on mood symptoms [35].

In the stratified analysis, the current study found that the proportion of high score group of total biological rhythm disorder increased with higher academic stages. We have tried to interpret the finding. In the middle and high school stages, behavioral and environmental factors (peer pressure, study burden, etc.) make students more inclined to sleep late and lack sleep on weekdays [36]. However, the overall sleep needs of adolescents remain constant [37], which may lead to sleep compensatory behavior at weekends and social jetlag related to the worsening of biorhythm disorders [38]. High school students, compared to middle school students, suffer more severe self-rating biological rhythm disorder due to the heavier stress and burden [39]. University students with more freedom in daily life are more likely to have access to mobile phones without restriction than middle and high school students who are not allowed to bring digital media such as smartphones to campus in China [40]. We also found that the proportion of high score group of digital media use dimension also increased with higher academic stages in the present study, which confirmed the above inference. Notably, although the proportion of high score group of activity rhythm disorders was significantly lower among university students possibly due to having more autonomy, the university students in general still had the highest proportion of high score group of total biological rhythm disorders. Similarly, at the high school phase, students from vocational schools do not face competitive study burden or college entrance examinations unlike students in senior high schools, and have a lower level of stress and higher usage of digital media than senior high school [41], therefore vocational school students have a higher proportion of high score group of total biological rhythm disorders. In the current study, junior high school students with high score were found to have the highest risk in suffering depression and anxiety symptoms among four academic stages. Junior high school students are at the onset of adolescence, and the sleep-wake cycle and melatonin rhythm begin to show a phase delay [42] which depends on the presence of gonadotropins and occurs simultaneously with sexual maturation [43, 44]. Moreover, exposure to nighttime light through lamps and luminous devices inhibits melatonin secretion and adolescents are more sensitive to light at night (23:00 to 24:00). Of note, the inhibitory effect is greater in the onset of adolescence [45].

Biological rhythms of organisms are controlled by circadian clocks, most obviously via the sleep-wake cycle, which can respond to external time and assist biological rhythms in regulating biological processes [46]. The suprachiasmatic nucleus (SCN) in the hypothalamus acts as the master pacemaker, setting the timing of rhythms by regulating neuronal activity, body temperature, and hormonal signals [47]. Circadian rhythms and sleep disruption may play a role in susceptibility to mood disorders [48, 49], of which the mechanisms involved is being studied. For example, disruption of circadian rhythms impacts endogenous melatonin homeostasis which is strongly associated with the onset, exacerbation, and recovery of depression symptoms [50]. Circadian rhythms disruption may also affect synaptic pruning and maturation of neural circuits during adolescence, leading to the development of psychological disorders [51]. Sleep-wake cycle is a primary part of biorhythm disorder and it’s also interrelated with other dimensions. For instance, UK National Diet and Nutrition Survey showed that scores of healthy eating patterns dropped when people slept 1 h and 45 minutes more on weekends than on weekdays [52]. Also, individuals presenting irregular sleep-wake cycles were more likely to present different times for meals and socialization [24]. Besides, using digital media before bedtime can affect sleep as well. Not only can the usage of digital media before bedtime delay sleep onset [53], but also the short wavelengths of light emitted from screens at night may suppress melatonin secretion to increase alertness [54], which will influence circadian rhythms. With the increasing popularity of electronic media in recent years, excessive electronic device use should be taken into account as an emerging risk factor. There was research that suggested that any harm caused by digital media use appears to come from the consumption of harmful content or healthy lifestyle substitutions with health effects, rather than just the frequent use of digital social media itself [55]. In the future, hopefully more research will focus on the use of electronic devices.

To the best of our knowledge, this is the first study to explore the association between self-rating biological rhythm disorders and depression and anxiety by academic stages, and one of the few studies on biological rhythm disorders among Chinese adolescents. Besides, unlike previous studies that assessed biorhythm disorders by BRIAN, the current study used the newly developed SBRDA, which not only evaluated from multiple dimensions but also was more suitable for Chinese adolescents in the contemporary, in order to make the assessment more accurate. Furthermore, this study was based on a large population covering 11–23 years old.

Despite these advantages, some limitations need to be noted. Firstly, as all data were self-reported by participants, there is inevitably information bias. However, the present study took some measures to minimize this problem. For example, we selected measurement tools with certain reliability and validity, ensured that students completed questionnaires independently in the classroom, and reviewed questionnaires for completeness promptly. Secondly, the cross-sectional study design can only explore correlations, hampering our ability to assess causal relationships. Thirdly, the sample for this study was collected from only one city in Henan Province of China, and the generalizability to the total population of China remains to be verified. Nevertheless, the fact that the city of Zhengzhou in Henan Province is located in central China mitigates this limitation to some extent. It should be also noted that the questionnaire used in the study was developed for Chinese adolescents, so its external validity abroad has yet to be verified.

## Conclusions

In this study, we found significant associations between self-rating biological rhythm disorders and both depression and anxiety symptoms. Junior high school students were found to have the highest risk in suffering depression and anxiety symptoms among four academic stages. We should also take discrepancy across academic stages into consideration in establishing public health strategies. Owing to the cross-sectional design of the current study, large-scale prospective studies are proposed to confirm these associations.

## Supplementary Information

**Additional file 1: Fig. S1.** Study participant flow diagram.

**Additional file 2: Table S1.** Sensitivity analysis. **Table S2.** The distribution of the score of the Self-Rating of Biological Rhythm Disorder for Adolescents (SBRDA). **Table S3.** Sample characteristics stratified by sleep rhythm disorder (*N* = 3693). **Table S4.** Sample characteristics stratified by activity rhythm disorder (*N* = 3693). **Table S5.** Sample characteristics stratified by eating habits rhythm disorder (*N* = 3693). **Table S6.** Sample characteristics stratified by digital media use (*N* = 3693). **Table S7.** Partial coefficients between self-rating biological rhythm disorders and Depression and anxiety symptoms. **Table S8.** Adjusted associations between self-rating biological rhythm disorders and depression symptoms. **Table S9.** Adjusted associations between self-rating biological rhythm disorders and anxiety symptoms.

## Data Availability

The datasets generated and/or analysed during the current study are not publicly available due to privacy/ ethical restrictions but are available from the corresponding author on reasonable request.

## Abbreviations

CI: confidence interval
OR: odd ratio
COVID-19: coronavirus disease 2019
SD: standard deviation
SPSS: Statistics Package for Social Science
M: middle score group
H: high score group
J: junior high school
V: vocational high school
S: senior high school
U: university
T: total
GAD-7: the 7-item Generalized Anxiety Disorder
PHQ-9: the Patient Health Questionnaire-9
BRIAN: the Biological Rhythms Interview of Assessment in Neuropsychiatry
SBRDA: the Self-Rating of Biological Rhythm Disorder for Adolescents.
