# Supplementary figures and images for: Multidimensional self-rating biological rhythm disorder and its association with depression and anxiety symptoms among adolescents aged 11–23 years: a school-based cross-sectional study from China
Source: BMC Psychiatry. 2022 Nov 14;22:700. doi: 10.1186/s12888-022-04354-8 (PMC9662778; doi:10.1186/s12888-022-04354-8)

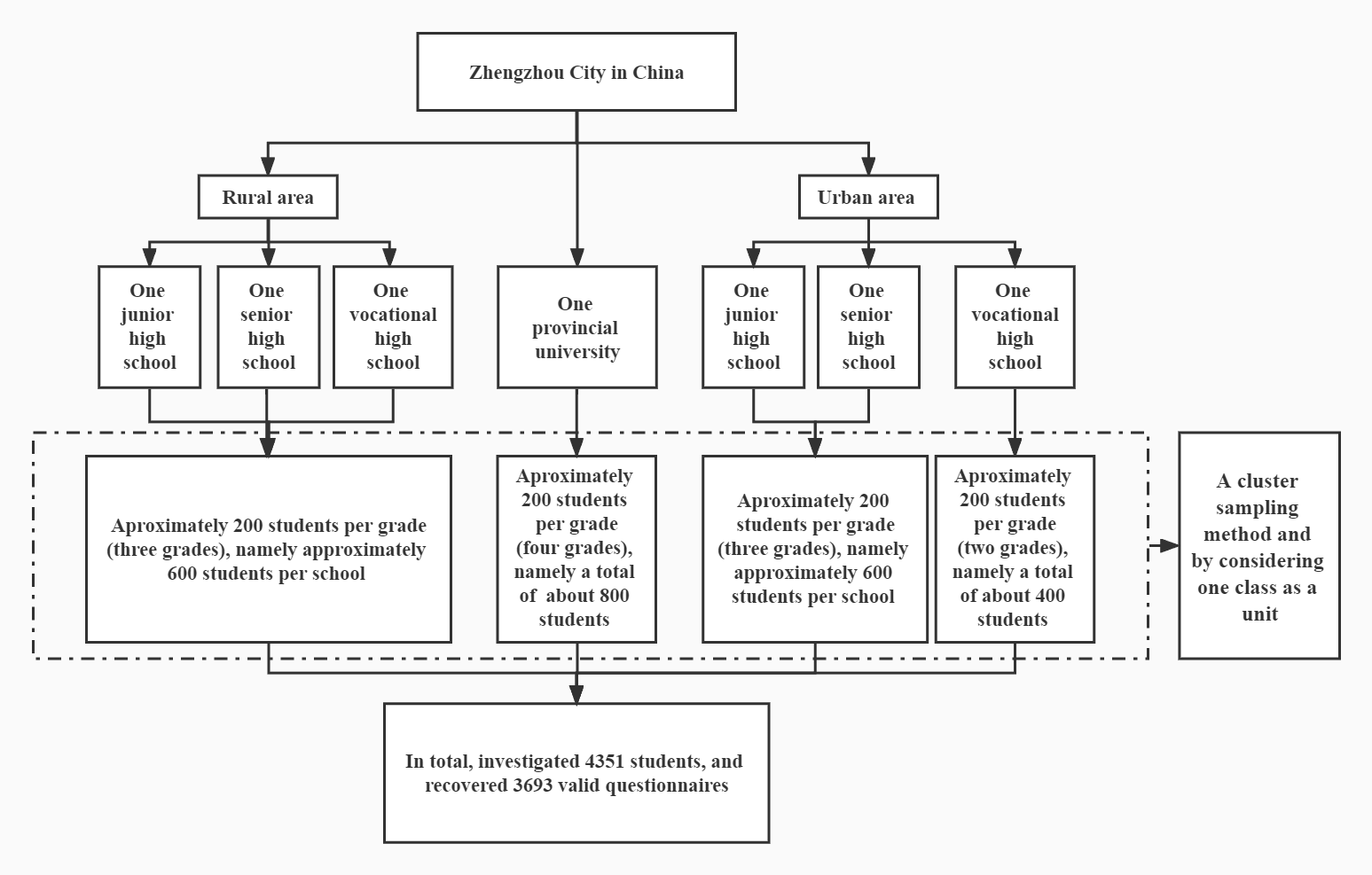

Supplement: Supplementary file 1 — Additional file 1: Fig. S1. Study participant flow diagram. [file 12888_2022_4354_MOESM1_ESM.jpg]
