# Supplementary material for: Multidimensional self-rating biological rhythm disorder and its association with depression and anxiety symptoms among adolescents aged 11–23 years: a school-based cross-sectional study from China
Source: BMC Psychiatry. 2022 Nov 14;22:700. doi: 10.1186/s12888-022-04354-8 (PMC9662778; doi:10.1186/s12888-022-04354-8)
Supplement: Supplementary file 2 — Additional file 2: Table S1. Sensitivity analysis. Table S2. The distribution of the score of the Self-Rating of Biological Rhythm Disorder for Adolescents (SBRDA). Table S3. Sample characteristics stratified by sleep rhythm disorder (N = 3693). Table S4. Sample characteristics stratified by activity rhythm disorder (N = 3693). Table S5. Sample characteristics stratified by eating habits rhythm disorder (N = 3693). Table S6. Sample characteristics stratified by digital media use (N = 3693). Table S7. Partial coefficients between self-rating biological rhythm disorders and Depression and anxiety symptoms. Table S8. Adjusted associations between self-rating biological rhythm disorders and depression symptoms. Table S9. Adjusted associations between self-rating biological rhythm disorders and anxiety symptoms. [file 12888_2022_4354_MOESM2_ESM.docx]

Table S1: Sensitivity analysis

| **Variables** | | **Initial samples** | **Included samples** | ***χ^2^ / t*** | ***P*** |
| --- | --- | --- | --- | --- | --- |
| Gender | Male | 2124(48.82%) | 1782(48.25%) | 0.554 | 0.457 |
|  | Female | 2203(50.63%) | 1911(51.75%) |  |  |
|  | Missing | 24(0.55%) |  |  |  |
| Academic stage | Junior high school | 1257(28.6%) | 1020(27.62%) | 4.977 | 0.173 |
|  | Vocational high school | 1036(23.8%) | 849(22.99%) |  |  |
|  | Senior high school | 1243(28.6%) | 1067(28.89%) |  |  |
|  | University | 815(18.7%) | 757(20.50%) |  |  |
| Residential areas | Rural | 2223(51.09%) | 1890(51.18%) | 0.002 | 0.961 |
|  | Urban | 2116(48.63%) | 1803(48.82%) |  |  |
|  | Missing | 12(0.28%) |  |  |  |

Table S2: The distribution of the score of the Self-Rating of Biological Rhythm Disorder for Adolescents (SBRDA)

| **Variables** | **Sleep rhythm**  **(Mean±SD)** | **Activity rhythm (Mean±SD)** | **Eating habits rhythm**  **(Mean±SD)** | **Digital media use**  **(Mean±SD)** | **Biological rhythm(total) (Mean±SD)** |
| --- | --- | --- | --- | --- | --- |
| **All** | 18.37±4.69 | 15.88±4.89 | 20.91±7.00 | 19.50±7.42 | 74.66±19.37 |
| **Gender** |  |  |  |  |  |
| Male | 17.84±4.87 | 15.73±4.98 | 19.91±6.95 | 18.89±7.41 | 72.38±19.65 |
| Female | 18.87±4.46 | 16.01±4.81 | 21.84±6.91 | 20.06±7.38 | 76.80±18.86 |
| **Grade** |  |  |  |  |  |
| Junior high school | 17.14±5.08 | 15.29±5.46 | 19.54±7.73 | 16.76±7.78 | 68.72±21.59 |
| Vocational high school | 17.88±4.76 | 16.19±5.00 | 21.26±7.08 | 20.39±6.86 | 75.72±19.74 |
| Senior high school | 19.76±4.19 | 16.57±4.52 | 21.32±6.26 | 19.17±7.21 | 76.82±17.04 |
| University | 18.64±4.16 | 15.36±4.28 | 21.81±6.60 | 22.64±6.33 | 78.45±16.95 |
| **Residential areas** |  |  |  |  |  |
| Rural | 17.99±4.47 | 16.33±4.75 | 21.14±6.71 | 20.27±7.14 | 75.74±18.67 |
| Urban | 18.78±4.88 | 15.40±4.99 | 20.67±7.28 | 18.69±7.62 | 73.54±20.01 |
| **Only child status** |  |  |  |  |  |
| No | 18.28±4.64 | 15.93±4.85 | 20.95±6.91 | 19.56±7.38 | 74.72±19.24 |
| Yes | 19.07±4.96 | 15.51±5.15 | 20.63±7.60 | 19.08±7.69 | 74.28±20.30 |
| **Father’s education** |  |  |  |  |  |
| Below elementary school | 18.38±4.14 | 16.20±4.46 | 21.41±7.34 | 21.13±7.43 | 77.11±19.53 |
| Elementary school | 18.24±4.61 | 16.32±5.01 | 21.15±6.93 | 20.31±7.44 | 76.03±19.46 |
| Junior high school | 18.15±4.52 | 16.07±4.85 | 20.95±6.79 | 19.68±7.13 | 74.87±18.80 |
| Senior high school or technical school | 18.59±4.92 | 15.61±4.89 | 20.94±7.13 | 18.85±7.46 | 73.99±19.55 |
| Junior college or above | 18.86±4.98 | 15.19±4.95 | 20.37±7.40 | 18.74±8.07 | 73.16±20.61 |
| **Mother’s education** |  |  |  |  |  |
| Below elementary school | 18.2±4.41 | 16.51±4.69 | 21.26±6.66 | 20.92±6.57 | 76.90±17.52 |
| Elementary school | 18.47±4.69 | 16.40±4.91 | 21.45±7.14 | 20.45±7.41 | 76.77±19.93 |
| Junior high school | 18.17±4.50 | 16.06±4.87 | 20.89±6.88 | 19.49±7.33 | 74.62±19.05 |
| Senior high school or technical school | 18.48±5.02 | 15.38±4.83 | 20.58±7.01 | 18.53±7.32 | 72.98±19.39 |
| Junior college or above | 18.83±4.87 | 14.97±4.99 | 20.54±7.33 | 18.87±8.10 | 73.22±20.34 |
| **Self-perceived family economy** |  |  |  |  |  |
| Under moderate | 18.64±4.42 | 16.68±4.9 | 22.19±6.97 | 21.55±7.32 | 79.07±18.80 |
| Moderate | 18.37±4.67 | 15.88±4.8 | 20.82±6.88 | 19.35±7.21 | 74.41±18.92 |
| Over moderate | 17.97±5.21 | 14.56±5.22 | 19.39±7.50 | 17.09±8.08 | 69.01±21.55 |
| **Self-perceived study burden** |  |  |  |  |  |
| Low | 17.44±5.16 | 14.67±5.63 | 20.03±8.49 | 18.46±8.73 | 70.60±23.65 |
| Medium | 17.92±4.52 | 15.51±4.74 | 20.31±6.76 | 19.14±7.19 | 72.89±18.77 |
| High | 19.27±4.77 | 16.67±4.92 | 22.05±7.03 | 20.25±7.55 | 78.24±19.19 |
| **The number of close friends** |  |  |  |  |  |
| 0 | 19.66±6.24 | 17.97±5.99 | 22.40±8.48 | 20.99±10.56 | 81.01±26.64 |
| 1-2 | 18.7±4.76 | 16.48±5.01 | 21.81±7.25 | 19.60±7.35 | 76.58±19.78 |
| 3-5 | 18.32±4.49 | 15.78±4.71 | 20.81±6.70 | 19.74±7.22 | 74.64±18.43 |
| >5 | 18.01±4.81 | 15.20±4.90 | 19.96±7.03 | 18.75±7.54 | 71.93±19.69 |

Table S3: Sample characteristics stratified by sleep rhythm disorder (N=3693)

| **Variables** | **N (%)** | **Sleep rhythm** | | | |
| --- | --- | --- | --- | --- | --- |
|  |  | **Low (%)** | **Middle (%)** | **High (%)** | ***χ^2^ / K-W*** |
| **Gender** |  |  |  |  |  |
| Male | 1782(48.25) | 526(29.5) | 846(47.5) | 410(23.0) | 29.35** |
| Female | 1911(51.75) | 421(22.0) | 963(50.4%) | 527(27.6) |  |
| **Academic stage** |  |  |  |  |  |
| Junior high school | 1020(27.62) | 376(36.9) | 440(43.1) | 204(20.0) | 159.47** |
| Vocational high school | 849(22.99) | 245(28.9) | 415(48.9) | 189(22.3) |  |
| Senior high school | 1067(28.89) | 162(15.2) | 546(51.2) | 359(33.6) |  |
| University | 757(20.50) | 164(21.7) | 408(53.9) | 185(24.4) |  |
| **Residential areas** |  |  |  |  |  |
| Rural | 1890(51.18) | 524(27.7) | 968(51.2) | 398(21.1) | 38.88** |
| Urban | 1803(48.82) | 423(23.5) | 841(46.6) | 539(29.9) |  |
| **Only child status** |  |  |  |  |  |
| No | 3240(87.73) | 847(26.1) | 1604(49.5) | 789(24.4) | 14.87** |
| Yes | 453(12.27) | 100(22.1) | 205(45.3) | 148(32.7) |  |
| **Father’s education** |  |  |  |  |  |
| Below elementary school | 133(3.60) | 33(24.8) | 75(56.4) | 25(18.8) | 16.36** |
| Elementary school | 509(13.78) | 134(26.3) | 255(50.1) | 120(23.6) |  |
| Junior high school | 1678(45.44) | 441(26.3) | 857(51.1) | 380(22.6) |  |
| Senior high school or technical school | 855(23.15) | 220(25.7) | 391(45.7) | 244(28.5) |  |
| Junior college or above | 518(14.03) | 119(23.0) | 231(44.6) | 168(32.4) |  |
| **Mother’s education** |  |  |  |  |  |
| Below elementary school | 300(8.12) | 86(28.7) | 148(49.3) | 66(22.0) | 12.70* |
| Elementary school | 658(17.82) | 161(24.5) | 326(49.5) | 171(26.0) |  |
| Junior high school | 1495(40.48) | 392(26.2) | 767(51.3) | 336(22.5) |  |
| Senior high school or technical school | 793(21.47) | 202(25.5) | 364(45.9) | 227(28.6) |  |
| Junior college or above | 447(12.10) | 106(23.7) | 204(45.6) | 137(30.6) |  |
| **Self-perceived family economy** |  |  |  |  |  |
| Under moderate | 649(17.57) | 143(22.0) | 347(53.5) | 159(24.5) | 2.99 |
| Moderate | 2656(71.92) | 674(25.4) | 1313(49.4) | 669(25.2) |  |
| Over moderate | 388(10.51) | 130(33.5) | 149(38.4) | 109(28.1) |  |
| **Self-perceived study burden** |  |  |  |  |  |
| Low | 183(4.96) | 70(38.3) | 69(37.7) | 44(24.0) | 63.33** |
| Medium | 2205(59.71) | 622(28.2) | 1104(50.1) | 479(21.7) |  |
| High | 1305(35.34) | 255(19.5) | 636(48.7) | 414(31.7) |  |
| **The number of close friends** |  |  |  |  |  |
| 0 | 73(1.98) | 15(20.5) | 24(32.9) | 34(46.6) | 18.19** |
| 1-2 | 1000(27.08) | 234(23.4) | 488(48.8) | 278(27.8) |  |
| 3-5 | 1768(47.87) | 455(25.7) | 892(50.5) | 421(23.8) |  |
| >5 | 852(23.07) | 243(28.5) | 405(47.5) | 204(23.9) |  |
| *p < 0.05; **p < 0.01 | | | | | |

Table S4: Sample characteristics stratified by activity rhythm disorder (N=3693)

| **Variables** | **N (%)** | **Activity rhythm** | | | |
| --- | --- | --- | --- | --- | --- |
|  |  | **Low (%)** | **Middle (%)** | **High (%)** | ***χ^2^ / K-W*** |
| **Gender** |  |  |  |  |  |
| Male | 1782(48.25) | 500(28.1) | 895(50.2) | 387(21.7) | 15.14** |
| Female | 1911(51.75) | 438(22.9) | 1067(55.8) | 406(21.2) |  |
| **Academic stage** |  |  |  |  |  |
| Junior high school | 1020(27.62) | 346(33.9) | 457(44.8) | 217(21.3) | 90.19** |
| Vocational high school | 849(22.99) | 196(23.1) | 452(53.2) | 201(23.7) |  |
| Senior high school | 1067(28.89) | 203(19.0) | 603(56.5) | 261(24.5) |  |
| University | 757(20.50) | 193(25.5) | 450(59.4) | 114(15.1) |  |
| **Residential areas** |  |  |  |  |  |
| Rural | 1890(51.18) | 397(21.0) | 1055(55.8) | 438(23.2) | 39.93** |
| Urban | 1803(48.82) | 541(30.0) | 907(50.3) | 355(19.7) |  |
| **Only child status** |  |  |  |  |  |
| No | 3240(87.73) | 798(24.6) | 1754(54.1) | 688(21.2) | 11.92** |
| Yes | 453(12.27) | 140(30.9) | 208(45.9) | 105(23.2) |  |
| **Father’s education** |  |  |  |  |  |
| Below elementary school | 133(3.60) | 28(21.1) | 78(58.6) | 27(20.3) | 18.66** |
| Elementary school | 509(13.78) | 110(21.6) | 282(55.4) | 117(23.0) |  |
| Junior high school | 1678(45.44) | 402(24.0) | 894(53.3) | 382(22.8) |  |
| Senior high school or technical school | 855(23.15) | 231(27.0) | 453(53.0) | 171(20.0) |  |
| Junior college or above | 518(14.03) | 167(32.2) | 255(49.2) | 96(18.5) |  |
| **Mother’s education** |  |  |  |  |  |
| Below elementary school | 300(8.12) | 57(19.0) | 166(55.3) | 77(25.7) | 32.88** |
| Elementary school | 658(17.82) | 138(21.0) | 364(55.3) | 156(23.7) |  |
| Junior high school | 1495(40.48) | 365(24.4) | 808(54.0) | 322(21.5) |  |
| Senior high school or technical school | 793(21.47) | 222(28.0) | 415(52.3) | 156(19.7) |  |
| Junior college or above | 447(12.10) | 156(34.9) | 209(46.8) | 82(18.3) |  |
| **Self-perceived family economy** |  |  |  |  |  |
| Under moderate | 649(17.57) | 125(19.3) | 355(54.7) | 169(26.0) | 50.10** |
| Moderate | 2656(71.92) | 658(24.8) | 1436(54.1) | 562(21.2) |  |
| Over moderate | 388(10.51) | 155(39.9) | 171(44.1) | 62(16.0) |  |
| **Self-perceived study burden** |  |  |  |  |  |
| Low | 183(4.96) | 74(40.4) | 76(41.5) | 33(18.0) | 46.43** |
| Medium | 2205(59.71) | 600(27.2) | 1181(53.6) | 424(19.2) |  |
| High | 1305(35.34) | 264(20.2) | 705(54.0) | 336(25.7) |  |
| **The number of close friends** |  |  |  |  |  |
| 0 | 73(1.98) | 14(19.2) | 30(41.1) | 29(39.7) | 39.54** |
| 1-2 | 1000(27.08) | 219(21.9) | 521(52.1) | 260(26.0) |  |
| 3-5 | 1768(47.87) | 439(24.8) | 977(55.3) | 352(19.9) |  |
| >5 | 852(23.07) | 266(31.2) | 434(50.9) | 152(17.8) |  |
| *p < 0.05; **p < 0.01 | | | | | |

Table S5: Sample characteristics stratified by eating habits rhythm disorder (N=3693)

| **Variables** | **N (%)** | **Diet rhythm** | | | |
| --- | --- | --- | --- | --- | --- |
|  |  | **Low (%)** | **Middle (%)** | **High (%)** | ***χ^2^ / K-W*** |
| **Gender** |  |  |  |  |  |
| Male | 1782(48.25) | 570(32.0) | 856(48.0) | 356(20.0) | 55.48** |
| Female | 1911(51.75) | 416(21.8) | 988(51.7) | 507(26.5) |  |
| **Academic stage** |  |  |  |  |  |
| Junior high school | 1020(27.62) | 389(38.1) | 415(40.7) | 216(21.2) | 104.19** |
| Vocational high school | 849(22.99) | 211(24.9) | 426(50.2) | 212(25.0) |  |
| Senior high school | 1067(28.89) | 234(21.9) | 591(55.4) | 242(22.7) |  |
| University | 757(20.50) | 152(20.1) | 412(54.4) | 193(25.5) |  |
| **Residential areas** |  |  |  |  |  |
| Rural | 1890(51.18) | 455(24.1) | 999(52.9) | 436(23.1) | 16.77** |
| Urban | 1803(48.82) | 531(29.5) | 845(46.9) | 427(23.7) |  |
| **Only child status** |  |  |  |  |  |
| No | 3240(87.73) | 843(26.0) | 1641(50.6) | 756(23.3) | 7.31* |
| Yes | 453(12.27) | 143(31.6) | 203(44.8) | 107(23.6) |  |
| **Father’s education** |  |  |  |  |  |
| Below elementary school | 133(3.60) | 37(27.8) | 60(45.1) | 36(27.1) | 4.42 |
| Elementary school | 509(13.78) | 128(25.1) | 255(50.1) | 126(24.8) |  |
| Junior high school | 1678(45.44) | 433(25.8) | 862(51.4) | 383(22.8) |  |
| Senior high school or technical school | 855(23.15) | 226(26.4) | 427(49.9) | 202(23.6) |  |
| Junior college or above | 518(14.03) | 162(31.3) | 240(46.3) | 116(22.4) |  |
| **Mother’s education** |  |  |  |  |  |
| Below elementary school | 300(8.12) | 66(22.0) | 166(55.3) | 68(22.7) | 5.46 |
| Elementary school | 658(17.82) | 166(25.2) | 323(49.1) | 169(25.7) |  |
| Junior high school | 1495(40.48) | 390(26.1) | 769(51.4) | 336(22.5) |  |
| Senior high school or technical school | 793(21.47) | 225(28.4) | 380(47.9) | 188(23.7) |  |
| Junior college or above | 447(12.10) | 139(31.1) | 206(46.1) | 102(22.8) |  |
| **Self-perceived family economy** |  |  |  |  |  |
| Under moderate | 649(17.57) | 135(20.8) | 340(52.4) | 174(26.8) | 27.43** |
| Moderate | 2656(71.92) | 706(26.6) | 1338(50.4) | 612(23.0) |  |
| Over moderate | 388(10.51) | 145(37.4) | 166(42.8) | 77(19.8) |  |
| **Self-perceived study burden** |  |  |  |  |  |
| Low | 183(4.96) | 68(37.2) | 70(38.3) | 45(24.6) | 43.57** |
| Medium | 2205(59.71) | 643(29.2) | 1110(50.3) | 452(20.5) |  |
| High | 1305(35.34) | 275(21.1) | 664(50.9) | 366(28.0) |  |
| **The number of close friends** |  |  |  |  |  |
| 0 | 73(1.98) | 20(27.4) | 32(43.8) | 21(28.8) | 26.10** |
| 1-2 | 1000(27.08) | 231(23.1) | 491(49.1) | 278(27.8) |  |
| 3-5 | 1768(47.87) | 460(26.0) | 917(51.9) | 391(22.1) |  |
| >5 | 852(23.07) | 275(32.3) | 404(47.4) | 173(20.3) |  |
| *p < 0.05; **p < 0.01 | | | | | |

Table S6: Sample characteristics stratified by digital media use (N=3693)

| **Variables** | **N (%)** | **Electronic product use** | | | |
| --- | --- | --- | --- | --- | --- |
|  |  | **Low (%)** | **Middle (%)** | **High (%)** | ***χ^2^/ K-W*** |
| **Gender** |  |  |  |  |  |
| Male | 1782(48.25) | 513(28.8) | 915(51.3) | 354(19.9) | 20.56** |
| Female | 1911(51.75) | 450(23.5) | 983(51.4) | 478(25.0) |  |
| **Academic stage** |  |  |  |  |  |
| Junior high school | 1020(27.62) | 446(43.7) | 427(41.9) | 147(14.4) | 327.99** |
| Vocational high school | 849(22.99) | 160(18.8) | 484(57.0) | 205(24.1) |  |
| Senior high school | 1067(28.89) | 282(26.4) | 571(53.5) | 214(20.1) |  |
| University | 757(20.50) | 75(9.9) | 416(55.0) | 266(35.1) |  |
| **Residential areas** |  |  |  |  |  |
| Rural | 1890(51.18) | 396(21.0) | 1035(54.8) | 459(24.3) | 52.82** |
| Urban | 1803(48.82) | 567(31.4) | 863(47.9) | 373(20.7) |  |
| **Only child status** |  |  |  |  |  |
| No | 3240(87.73) | 827(25.5) | 1679(51.8) | 734(22.7) | 4.20 |
| Yes | 453(12.27) | 136(30.0) | 219(48.3) | 98(21.6) |  |
| **Father’s education** |  |  |  |  |  |
| Below elementary school | 133(3.60) | 22(16.5) | 76(57.1) | 35(26.3) | 24.10** |
| Elementary school | 509(13.78) | 111(21.8) | 264(51.9) | 134(26.3) |  |
| Junior high school | 1678(45.44) | 396(23.6) | 919(54.8) | 363(21.6) |  |
| Senior high school or technical school | 855(23.15) | 263(30.8) | 410(48.0) | 182(21.3) |  |
| Junior college or above | 518(14.03) | 171(33.0) | 229(44.2) | 118(22.8) |  |
| **Mother’s education** |  |  |  |  |  |
| Below elementary school | 300(8.12) | 45(15.0) | 179(59.7) | 76(25.3) | 32.52** |
| Elementary school | 658(17.82) | 147(22.3) | 340(51.7) | 171(26.0) |  |
| Junior high school | 1495(40.48) | 383(25.6) | 783(52.4) | 329(22.0) |  |
| Senior high school or technical school | 793(21.47) | 241(30.4) | 400(50.4) | 152(19.2) |  |
| Junior college or above | 447(12.10) | 147(32.9) | 196(43.8) | 104(23.3) |  |
| **Self-perceived family economy** |  |  |  |  |  |
| Under moderate | 649(17.57) | 112(17.3) | 343(52.9) | 194(29.9) | 75.75** |
| Moderate | 2656(71.92) | 685(25.8) | 1399(52.7) | 572(21.5) |  |
| Over moderate | 388(10.51) | 166(42.8) | 156(40.2) | 66(17.0) |  |
| **Self-perceived study burden** |  |  |  |  |  |
| Low | 183(4.96) | 70(38.3) | 72(39.3) | 41(22.4) | 29.99** |
| Medium | 2205(59.71) | 580(26.3) | 1172(53.2) | 453(20.5) |  |
| High | 1305(35.34) | 313(24.0) | 654(50.1) | 338(25.9) |  |
| **The number of close friends** |  |  |  |  |  |
| 0 | 73(1.98) | 22(30.1) | 28(38.4) | 23(31.5) | 9.17* |
| 1-2 | 1000(27.08) | 251(25.1) | 525(52.5) | 224(22.4) |  |
| 3-5 | 1768(47.87) | 439(24.8) | 912(51.6) | 417(23.6) |  |
| >5 | 852(23.07) | 251(29.5) | 433(50.8) | 168(19.7) |  |
| *p < 0.05; **p < 0.01 | | | | | |

Table S7: Partial coefficients between self-rating biological rhythm disorders and Depression and anxiety symptoms

| **Variables** | **Sleep rhythm** | **Activity rhythm** | **Eating habits rhythm** | **Digital media use** | **Biological rhythm (Total)** |
| --- | --- | --- | --- | --- | --- |
| **Depression symptoms** | 0.313** | 0.461** | 0.413** | 0.371** | 0.485** |
| **Anxiety symptoms** | 0.257** | 0.387** | 0.364** | 0.324** | 0.417** |
| *p < 0.05; **p < 0.01 | | | | | |

Table S8: Adjusted associations between self-rating biological rhythm disorders and depression symptoms

| **Variables** | **Academic stage** | **Score** | **Depression symptoms** | | | | | | | | |
| --- | --- | --- | --- | --- | --- | --- | --- | --- | --- | --- | --- |
|  |  |  | **cOR** | **Lower** | **Upper** | ***P*** |  | **aOR** | **Lower** | **Upper** | ***P*** |
| **Sleep rhythm** | **Junior high school** | Low |  |  |  |  |  |  |  |  |  |
|  |  | Middle | 2.43 | 1.79 | 3.29 | <0.01** |  | 2.41 | 1.74 | 3.35 | <0.01** |
|  |  | High | 5.00 | 3.46 | 7.22 | <0.01** |  | 4.72 | 3.15 | 7.06 | <0.01** |
|  | **Vocational high school** | Low |  |  |  |  |  |  |  |  |  |
|  |  | Middle | 3.14 | 2.19 | 4.49 | <0.01** |  | 3.46 | 2.35 | 5.08 | <0.01** |
|  |  | High | 6.91 | 4.51 | 10.58 | <0.01** |  | 7.93 | 5.00 | 12.56 | <0.01** |
|  | **Senior high school** | Low |  |  |  |  |  |  |  |  |  |
|  |  | Middle | 2.39 | 1.64 | 3.50 | <0.01** |  | 2.59 | 1.74 | 3.88 | <0.01** |
|  |  | High | 4.11 | 2.75 | 6.14 | <0.01** |  | 4.72 | 3.07 | 7.28 | <0.01** |
|  | **University** | Low |  |  |  |  |  |  |  |  |  |
|  |  | Middle | 2.06 | 1.37 | 3.10 | <0.01** |  | 1.95 | 1.27 | 3.00 | <0.01** |
|  |  | High | 4.45 | 2.80 | 7.05 | <0.01** |  | 4.57 | 2.79 | 7.49 | <0.01** |
|  | **Total** | Low |  |  |  |  |  |  |  |  |  |
|  |  | Middle | 2.57 | 2.16 | 3.06 | <0.01** |  | 2.56 | 2.13 | 3.08 | <0.01** |
|  |  | High | 5.13 | 4.21 | 6.26 | <0.01** |  | 5.27 | 4.27 | 6.51 | <0.01** |
| **Activity rhythm** | **Junior high school** | Low |  |  |  |  |  |  |  |  |  |
|  |  | Middle | 4.80 | 3.34 | 6.91 | <0.01** |  | 4.32 | 2.93 | 6.36 | <0.01** |
|  |  | High | 21.77 | 13.97 | 33.92 | <0.01** |  | 19.34 | 12.06 | 31.00 | <0.01** |
|  | **Vocational high school** | Low |  |  |  |  |  |  |  |  |  |
|  |  | Middle | 4.26 | 2.78 | 6.53 | <0.01** |  | 4.20 | 2.70 | 6.53 | <0.01** |
|  |  | High | 12.81 | 7.85 | 20.90 | <0.01** |  | 12.83 | 7.73 | 21.32 | <0.01** |
|  | **Senior high school** | Low |  |  |  |  |  |  |  |  |  |
|  |  | Middle | 2.74 | 1.92 | 3.93 | <0.01** |  | 2.65 | 1.82 | 3.87 | <0.01** |
|  |  | High | 13.60 | 8.70 | 21.26 | <0.01** |  | 12.48 | 7.82 | 19.93 | <0.01** |
|  | **University** | Low |  |  |  |  |  |  |  |  |  |
|  |  | Middle | 4.49 | 2.93 | 6.88 | <0.01** |  | 4.49 | 2.86 | 7.05 | <0.01** |
|  |  | High | 9.30 | 5.41 | 16.00 | <0.01** |  | 9.81 | 5.54 | 17.40 | <0.01** |
|  | **Total** | Low |  |  |  |  |  |  |  |  |  |
|  |  | Middle | 4.09 | 3.37 | 4.96 | <0.01** |  | 3.90 | 3.19 | 4.77 | <0.01** |
|  |  | High | 14.86 | 11.75 | 18.80 | <0.01** |  | 13.93 | 10.92 | 17.77 | <0.01** |
| **Eating habits rhythm** | **Junior high school** | Low |  |  |  |  |  |  |  |  |  |
|  |  | Middle | 4.28 | 3.06 | 6.01 | <0.01** |  | 3.78 | 2.64 | 5.41 | <0.01** |
|  |  | High | 17.20 | 11.35 | 26.07 | <0.01** |  | 14.90 | 9.58 | 23.18 | <0.01** |
|  | **Vocational high school** | Low |  |  |  |  |  |  |  |  |  |
|  |  | Middle | 3.03 | 2.06 | 4.44 | <0.01** |  | 3.53 | 2.35 | 5.33 | <0.01** |
|  |  | High | 7.54 | 4.87 | 11.68 | <0.01** |  | 8.56 | 5.35 | 13.70 | <0.01** |
|  | **Senior high school** | Low |  |  |  |  |  |  |  |  |  |
|  |  | Middle | 3.03 | 2.17 | 4.24 | <0.01** |  | 3.24 | 2.26 | 4.64 | <0.01** |
|  |  | High | 8.42 | 5.58 | 12.71 | <0.01** |  | 8.96 | 5.76 | 13.92 | <0.01** |
|  | **University** | Low |  |  |  |  |  |  |  |  |  |
|  |  | Middle | 2.98 | 1.90 | 4.67 | <0.01** |  | 2.88 | 1.80 | 4.61 | <0.01** |
|  |  | High | 5.99 | 3.65 | 9.84 | <0.01** |  | 5.56 | 3.31 | 9.36 | <0.01** |
|  | **Total** | Low |  |  |  |  |  |  |  |  |  |
|  |  | Middle | 3.47 | 2.89 | 4.16 | <0.01** |  | 3.43 | 2.83 | 4.15 | <0.01** |
|  |  | High | 9.28 | 7.50 | 11.49 | <0.01** |  | 9.07 | 7.25 | 11.34 | <0.01** |
| **Digital media use** | **Junior high school** | Low |  |  |  |  |  |  |  |  |  |
|  |  | Middle | 3.62 | 2.68 | 4.88 | <0.01** |  | 3.61 | 2.58 | 5.06 | <0.01** |
|  |  | High | 10.95 | 7.10 | 16.89 | <0.01** |  | 12.17 | 7.54 | 19.64 | <0.01** |
|  | **Vocational high school** | Low |  |  |  |  |  |  |  |  |  |
|  |  | Middle | 3.11 | 2.02 | 4.79 | <0.01** |  | 3.60 | 2.28 | 5.68 | <0.01** |
|  |  | High | 8.20 | 5.04 | 13.35 | <0.01** |  | 9.59 | 5.71 | 16.13 | <0.01** |
|  | **Senior high school** | Low |  |  |  |  |  |  |  |  |  |
|  |  | Middle | 2.42 | 1.79 | 3.26 | <0.01** |  | 2.52 | 1.83 | 3.48 | <0.01** |
|  |  | High | 5.32 | 3.61 | 7.83 | <0.01** |  | 5.38 | 3.56 | 8.11 | <0.01** |
|  | **University** | Low |  |  |  |  |  |  |  |  |  |
|  |  | Middle | 2.58 | 1.37 | 4.85 | <0.01** |  | 2.50 | 1.28 | 4.88 | <0.01** |
|  |  | High | 6.46 | 3.39 | 12.31 | <0.01** |  | 6.30 | 3.18 | 12.49 | <0.01** |
|  | **Total** | Low |  |  |  |  |  |  |  |  |  |
|  |  | Middle | 2.74 | 2.30 | 3.26 | <0.01** |  | 3.05 | 2.53 | 3.69 | <0.01** |
|  |  | High | 6.44 | 5.23 | 7.92 | <0.01** |  | 7.37 | 5.87 | 9.25 | <0.01** |
| **Biological rhythm (total)** | **Junior high school** | Low |  |  |  |  |  |  |  |  |  |
|  |  | Middle | 4.90 | 3.51 | 6.83 | <0.01** |  | 4.73 | 3.31 | 6.76 | <0.01** |
|  |  | High | 22.06 | 14.27 | 34.11 | <0.01** |  | 20.58 | 12.89 | 32.88 | <0.01** |
|  | **Vocational high school** | Low |  |  |  |  |  |  |  |  |  |
|  |  | Middle | 3.62 | 2.39 | 5.48 | <0.01** |  | 4.01 | 2.58 | 6.22 | <0.01** |
|  |  | High | 12.33 | 7.84 | 19.41 | <0.01** |  | 13.76 | 8.45 | 22.39 | <0.01** |
|  | **Senior high school** | Low |  |  |  |  |  |  |  |  |  |
|  |  | Middle | 3.80 | 2.62 | 5.50 | <0.01** |  | 3.85 | 2.61 | 5.66 | <0.01** |
|  |  | High | 13.90 | 9.08 | 21.26 | <0.01** |  | 13.21 | 8.49 | 20.56 | <0.01** |
|  | **University** | Low |  |  |  |  |  |  |  |  |  |
|  |  | Middle | 4.77 | 2.64 | 8.63 | <0.01** |  | 4.47 | 2.42 | 8.25 | <0.01** |
|  |  | High | 13.60 | 7.36 | 25.12 | <0.01** |  | 12.41 | 6.56 | 23.47 | <0.01** |
|  | **Total** | Low |  |  |  |  |  |  |  |  |  |
|  |  | Middle | 4.15 | 3.41 | 5.05 | <0.01** |  | 4.33 | 3.53 | 5.32 | <0.01** |
|  |  | High | 14.13 | 11.33 | 17.62 | <0.01** |  | 14.38 | 11.38 | 18.16 | <0.01** |
| *p < 0.05; **p < 0.01 | | | | | | | | | | | |

Table S9: Adjusted associations between self-rating biological rhythm disorders and anxiety symptoms

| **Dimensions** | **Academic stage** | **Score** | **Anxiety symptoms** | | | | | | | | |
| --- | --- | --- | --- | --- | --- | --- | --- | --- | --- | --- | --- |
|  |  |  | **cOR** | **Lower** | **Upper** | ***P*** |  | **aOR** | **Lower** | **Upper** | ***P*** |
| **Sleep rhythm** | **Junior high school** | Low |  |  |  |  |  |  |  |  |  |
|  |  | Middle | 2.04 | 1.49 | 2.79 | <0.01** |  | 1.97 | 1.41 | 2.75 | <0.01** |
|  |  | High | 4.35 | 3.01 | 6.29 | <0.01** |  | 4.12 | 2.76 | 6.15 | <0.01** |
|  | **Vocational high school** | Low |  |  |  |  |  |  |  |  |  |
|  |  | Middle | 2.84 | 1.92 | 4.21 | <0.01** |  | 3.06 | 2.01 | 4.66 | <0.01** |
|  |  | High | 5.76 | 3.70 | 8.97 | <0.01** |  | 6.29 | 3.91 | 10.12 | <0.01** |
|  | **Senior high school** | Low |  |  |  |  |  |  |  |  |  |
|  |  | Middle | 1.68 | 1.14 | 2.47 | <0.01** |  | 1.65 | 1.09 | 2.49 | 0.018* |
|  |  | High | 3.19 | 2.13 | 4.78 | <0.01** |  | 3.31 | 2.13 | 5.12 | <0.01** |
|  | **University** | Low |  |  |  |  |  |  |  |  |  |
|  |  | Middle | 1.38 | 0.91 | 2.10 | 0.127 |  | 1.29 | 0.83 | 2.00 | 0.25 |
|  |  | High | 2.78 | 1.76 | 4.42 | <0.01** |  | 2.73 | 1.67 | 4.46 | <0.01** |
|  | **Total** | Low |  |  |  |  |  |  |  |  |  |
|  |  | Middle | 1.99 | 1.66 | 2.39 | <0.01** |  | 1.95 | 1.61 | 2.36 | <0.01** |
|  |  | High | 4.03 | 3.29 | 4.92 | <0.01** |  | 3.97 | 3.21 | 4.91 | <0.01** |
| **Activity rhythm** | **Junior high school** | Low |  |  |  |  |  |  |  |  |  |
|  |  | Middle | 4.36 | 2.98 | 6.39 | <0.01** |  | 3.86 | 2.59 | 5.75 | <0.01** |
|  |  | High | 15.09 | 9.78 | 23.28 | <0.01** |  | 12.64 | 8.01 | 19.94 | <0.01** |
|  | **Vocational high school** | Low |  |  |  |  |  |  |  |  |  |
|  |  | Middle | 4.67 | 2.80 | 7.80 | <0.01** |  | 4.76 | 2.80 | 8.10 | <0.01** |
|  |  | High | 13.24 | 7.64 | 22.96 | <0.01** |  | 14.66 | 8.22 | 26.14 | <0.01** |
|  | **Senior high school** | Low |  |  |  |  |  |  |  |  |  |
|  |  | Middle | 2.68 | 1.81 | 3.95 | <0.01** |  | 2.58 | 1.71 | 3.89 | <0.01** |
|  |  | High | 9.82 | 6.33 | 15.25 | <0.01** |  | 8.99 | 5.63 | 14.34 | <0.01** |
|  | **University** | Low |  |  |  |  |  |  |  |  |  |
|  |  | Middle | 3.09 | 1.99 | 4.80 | <0.01** |  | 3.11 | 1.96 | 4.94 | <0.01** |
|  |  | High | 6.28 | 3.66 | 4.80 | <0.01** |  | 6.47 | 3.66 | 11.45 | <0.01** |
|  | **Total** | Low |  |  |  |  |  |  |  |  |  |
|  |  | Middle | 3.62 | 2.94 | 4.47 | <0.01** |  | 3.53 | 2.84 | 4.38 | <0.01** |
|  |  | High | 11.18 | 8.82 | 14.17 | <0.01** |  | 10.52 | 8.22 | 13.46 | <0.01** |
| **Eating habits rhythm** | **Junior high school** | Low |  |  |  |  |  |  |  |  |  |
|  |  | Middle | 3.59 | 2.52 | 5.10 | <0.01** |  | 3.04 | 2.11 | 4.38 | <0.01** |
|  |  | High | 13.51 | 8.99 | 20.29 | <0.01** |  | 11.12 | 7.27 | 17.02 | <0.01** |
|  | **Vocational high school** | Low |  |  |  |  |  |  |  |  |  |
|  |  | Middle | 2.50 | 1.64 | 3.82 | <0.01** |  | 2.93 | 1.86 | 4.61 | <0.01** |
|  |  | High | 7.04 | 4.44 | 11.15 | <0.01** |  | 8.52 | 5.17 | 14.05 | <0.01** |
|  | **Senior high school** | Low |  |  |  |  |  |  |  |  |  |
|  |  | Middle | 2.67 | 1.87 | 3.81 | <0.01** |  | 2.79 | 1.90 | 4.10 | <0.01** |
|  |  | High | 7.56 | 5.00 | 11.44 | <0.01** |  | 7.86 | 5.01 | 12.32 | <0.01** |
|  | **University** | Low |  |  |  |  |  |  |  |  |  |
|  |  | Middle | 2.57 | 1.59 | 4.16 | <0.01** |  | 2.58 | 1.57 | 4.26 | <0.01** |
|  |  | High | 4.66 | 2.77 | 7.84 | <0.01** |  | 4.18 | 2.44 | 7.19 | <0.01** |
|  | **Total** | Low |  |  |  |  |  |  |  |  |  |
|  |  | Middle | 2.92 | 2.40 | 3.55 | <0.01** |  | 2.93 | 2.39 | 3.60 | <0.01** |
|  |  | High | 7.83 | 6.30 | 9.74 | <0.01** |  | 7.75 | 6.16 | 9.75 | <0.01** |
| **Digital media use** | **Junior high school** | Low |  |  |  |  |  |  |  |  |  |
|  |  | Middle | 3.85 | 2.82 | 5.27 | <0.01** |  | 3.79 | 2.69 | 5.34 | <0.01** |
|  |  | High | 7.38 | 4.88 | 11.15 | <0.01** |  | 7.32 | 4.67 | 11.49 | <0.01** |
|  | **Vocational high school** | Low |  |  |  |  |  |  |  |  |  |
|  |  | Middle | 3.24 | 1.95 | 5.37 | <0.01** |  | 3.82 | 2.24 | 6.50 | <0.01** |
|  |  | High | 8.94 | 5.19 | 15.41 | <0.01** |  | 10.56 | 5.92 | 18.84 | <0.01** |
|  | **Senior high school** | Low |  |  |  |  |  |  |  |  |  |
|  |  | Middle | 1.86 | 1.37 | 2.54 | <0.01** |  | 1.99 | 1.42 | 2.78 | <0.01** |
|  |  | High | 3.98 | 2.73 | 5.80 | <0.01** |  | 4.29 | 2.84 | 6.46 | <0.01** |
|  | **University** | Low |  |  |  |  |  |  |  |  |  |
|  |  | Middle | 3.09 | 1.44 | 6.63 | <0.01** |  | 3.12 | 1.41 | 6.92 | <0.01** |
|  |  | High | 7.77 | 3.59 | 16.80 | <0.01** |  | 7.91 | 3.54 | 17.67 | <0.01** |
|  | **Total** | Low |  |  |  |  |  |  |  |  |  |
|  |  | Middle | 2.46 | 2.04 | 2.97 | <0.01** |  | 2.88 | 2.36 | 3.53 | <0.01** |
|  |  | High | 5.33 | 4.32 | 6.59 | <0.01** |  | 6.49 | 5.14 | 8.20 | <0.01** |
| **Biological rhythm (Total)** | **Junior high school** | Low |  |  |  |  |  |  |  |  |  |
|  |  | Middle | 4.93 | 3.46 | 7.01 | <0.01** |  | 4.64 | 3.21 | 6.72 | <0.01** |
|  |  | High | 16.15 | 10.63 | 24.55 | <0.01** |  | 13.95 | 8.96 | 21.72 | <0.01** |
|  | **Vocational high school** | Low |  |  |  |  |  |  |  |  |  |
|  |  | Middle | 3.41 | 2.11 | 5.49 | <0.01** |  | 3.80 | 2.30 | 6.29 | <0.01** |
|  |  | High | 11.07 | 6.76 | 18.14 | <0.01** |  | 12.78 | 7.51 | 21.74 | <0.01** |
|  | **Senior high school** | Low |  |  |  |  |  |  |  |  |  |
|  |  | Middle | 3.17 | 2.13 | 4.72 | <0.01** |  | 3.15 | 2.08 | 4.78 | <0.01** |
|  |  | High | 10.97 | 7.13 | 16.86 | <0.01** |  | 10.38 | 6.61 | 16.32 | <0.01** |
|  | **University** | Low |  |  |  |  |  |  |  |  |  |
|  |  | Middle | 3.90 | 2.07 | 7.36 | <0.01** |  | 4.05 | 2.10 | 7.80 | <0.01** |
|  |  | High | 9.74 | 5.11 | 18.57 | <0.01** |  | 9.06 | 4.63 | 17.71 | <0.01** |
|  | **Total** | Low |  |  |  |  |  |  |  |  |  |
|  |  | Middle | 3.72 | 3.01 | 4.61 | <0.01** |  | 3.99 | 3.19 | 4.98 | <0.01** |
|  |  | High | 11.16 | 8.89 | 14.01 | <0.01** |  | 11.63 | 9.14 | 14.81 | <0.01** |
| *p < 0.05; **p < 0.01 | | | | | | | | | | | |
